# Die Rolle der HabilitandInnen in der chirurgischen Lehre

**DOI:** 10.1007/s00104-021-01406-9

**Published:** 2021-04-14

**Authors:** Christoph Paasch, Carl Meißner, Frank Meyer

**Affiliations:** 1 Klinik für Allgemein‑, Viszeral-, Gefäß- und Transplantationschirurgie, Universitätsklinikum Magdeburg A.ö.R., Leipziger Str. 44, 39120 Magdeburg, Deutschland; 2 Klinik für Allgemein‑ und Viszeralchirurgie, Klinikum Magdeburg GmbH, Birkenallee 122, 39130 Magdeburg, Deutschland

**Keywords:** Humanmedizinstudium, Habilitation, Chirurgische Ausbildung, Akademische Graduierung, Lehrambition, Study of human medicine, Habilitation, Surgical education/training, Academic graduation, Teaching ambition

## Abstract

**Ziel:**

Die kompakte Übersicht skizziert die Verantwortung und das breite Aufgabenfeld der/s HabilitandIn in der chirurgischen Lehre im Rahmen des Humanmedizinstudiums.

**Methode:**

Narrative Kurzübersicht auf der Basis gewonnener individueller und einrichtungsspezifischer Lehrerfahrungen.

**Ergebnisse (Eckpunkte):**

Rolle der/des HabilitandIn in (Auswahl): (i) Abgrenzung zum nichthabilitierten ärztlichen Kollegen: Die im Rahmen des Habilitationsvorhabens gesammelten administrativen und wissenschaftlichen als auch Lehrerfahrungen lassen den/die HabilitandIn als geeigneteren, motivierteren und kompetenteren Promotionsverantwortlichen und -betreuer im Gegensatz zu Kollegen, die sich nicht habilitiert haben oder habilitieren möchten, erscheinen. (ii) Abhängigkeit medizinischer Disziplinen: Die Rolle, die in der Lehre eingenommen werden kann, ist durchaus stark fachabhängig. So bestehen die Möglichkeit und die Pflicht, in chirurgischen Fächern neben theoretischem Wissen stets auch kompetente praktische Fertigkeiten zu vermitteln. (iii) Universitäten und nichtuniversitäre Krankenhäuser: In nichtuniversitären (Lehr‑)Krankenhäusern können die/der chirurgische HabilitandIn die Aufgaben der chirurgischen Lehre durch eine komplette PJ- und Famulaturbetreuung erfüllen. An universitären Kliniken steht das Abhalten von Lehrveranstaltungen eher im Vordergrund.

**Schlussfolgerung:**

Die/der HabilitandIn spielt in der chirurgischen Lehre eine zentrale Rolle. Ein breites Aufgabenspektrum mit Abhaltung und Gestaltung von Vorlesungen, Seminaren, Blockpraktika bis hin zu direkter studentischer Betreuung im Rahmen der Famulatur, des Praktischen Jahrs und der Promotion kann von der Habilitandin/vom Habilitand suffizient erfüllt werden neben der ideenreichen Gestaltung fakultativer Lehrkonzepte.

## Abstract (en)

**Objective:**

This compact overview outlines the responsibilities and broad field of tasks of a post-doctoral fellow in surgical teaching within the framework of studies in human medicine.

**Method:**

Narrative short review based on own individual and institutional teaching experiences.

**Results:**

Role of the post-doctoral fellow (selection): (i) due to the planned (sub)specialization by the respective habilitation project and because of the accumulated (administrative and scientific as well as teaching) experience within the framework of this scientific graduation, the post-doctoral fellow appears to be a more suitable, motivated and competent doctoral manager and supervisor compared to colleagues who do not intend to get the habilitation. (ii) Dependence of medical disciplines: the role that can be taken in teaching is highly dependent on the subject matter and discipline. Thus, there is the possibility and the duty to always mediate competent practical skills in surgical subjects in addition to theoretical knowledge and to pay strict attention to their ready to use acquisition in preparation for clinical/surgical practice. (iii) In nonuniversity (teaching) hospitals, the surgical post-doctoral fellow can fulfill the tasks of surgical teaching through a complete internship and clerkship care. In university clinics the focus is more on holding lectures in addition to internship and clerkship tutorials.

**Conclusion:**

The post-doctoral fellow plays a central role in surgical teaching. A wide range of tasks with holding and construction of lectures, seminars and block internships up to direct student support within the framework of the clerkship, the practical year and the support of a dissertation can be sufficiently fulfilled by the post-doctoral fellow in addition to the development of facultative teaching concepts full of ideas.

## Hintergrund

Die Habilitation als höchste akademische Graduierung nimmt ebenso eine herausragende Stellung in der akademischen Lehre wie auch im Humanmedizinstudium ein. Dieser Rolle im Lehralltag gebührend Rechnung zu tragen, ist mit einem hohen und nachhaltigen Anspruch verbunden, dem sich die/der HabilitandIn als fast unabdingbar einzustufende Anforderung bereits früh stellen und würdig erweisen muss. Dabei hat sich neben stark traditionellen und überlieferten, da wohl bewährten Aspekten der Konstanz auch eine durchaus auffällig beobacht- und nachvollziehbare Entwicklung ergeben.

Ziel: Diese kompakte narrative Kurzübersicht soll sowohl die Rolle der HabilitandInnen als auch andeutungsweise mit Ausblick auf die Habilitation jene der/des bereits Habilitierten im breitgefächerten, anspruchsvollen und herausfordernden Themen‑, Methoden- und individuell angepassten Vorgehensspektrum in der chirurgischen Lehre mit diversen interdisziplinären und fächerspezifischen Querverweisen inklusive innovativer Ansätze illustrieren.

## Methode

Auf der Basis gewonnener individueller und einrichtungsspezifischer Lehrerfahrungen und selektiver Referenzen aus der einschlägigen medizinisch-wissenschaftlichen Literatur, z. B. PubMed und Google Scholar wurde eine narrative Kurzübersicht erstellt. Folgende Schlüsselwörter wurden hierbei benutzt: Habilitandin, Habilitand, Habilitierte(r), Humanmedizinstudium, chirurgische Lehre, chirurgische Ausbildung.

## Eckpunkte

### Definition der Habilitation

Der Begriff Habilitation leitet sich vom mittellateinischen Begriff *habilitatio* ab. Dieses Wort stammt wiederum vom Verb *habilitare *und bedeutet: befähigen, geeignet machen.

In der Bundesrepublik Deutschland ist die Habilitation die höchstrangige Hochschulprüfung. Diese Prüfung dient dazu, die Lehrbefähigung (lateinisch: *facultas docendi*) der HabilitandInnen in einem wissenschaftlichen Fach festzustellen (lateinisch: *venia legendi* [Erlaubnis vorzulesen]) und zu prüfen, ob die/der HabilitandIn das jeweilige Fach in voller Breite in Forschung und Lehre vertreten kann. Laut statistischem Bundesamt wurden 2019 fächerübergreifend 1529 Habilitationen durchgeführt. Ein Großteil dieser Arbeiten entfielen auf das Fach der Humanmedizin [1].

### Historie der Habilitation im europäischen und deutschsprachigen Raum

In Zeiten der mittelalterlichen und frühneuzeitlichen Universitäten war die Habilitation weitgehend unbekannt. Die Promotion hatte hier den Stellenwert der höchsten akademischen Ausbildung bzw. Graduierung; die sog. *disputatio* war die Regel. Erst im Laufe des 19. Jahrhunderts setzte sich die Habilitation als Eingangsvoraussetzung für die Professur und die akademische Laufbahn an allen deutschen Universitäten verbindlich durch [2, 3].

Im deutschen medizinischen Hochschulwesen beteiligen sich HabilitandInnen aktiv und vorrangig als auch gemäß ihrer akademischen Qualifikation und nicht zuletzt der damit zugedachten Rolle (wie auch länderspezifisch vorgegeben) neben den diversen Vertretern der anderen hierarchischen klinischen Struktur- und Lehrebenen an der Ausbildung von Studierenden der Humanmedizin.

### HabilitandIn vs. Habilitierte(r)

Während die/der chirurgische HabilitandIn eine akademische Lehrbefähigung und einen damit assoziierten Lehrauftrag (erst) anstrebt, hat die/der Habilitierte diese Hürde bereits genommen. Beiden Phasen im Habilitationsverfahren/-abschluss gleich eigen sollten die bestehende Einsicht und Motivation in anhaltend zu leistender chirurgischer Lehre in ihrer breit gefächerten Facettierung, ob kurrikular oder fakultativ, sein. Nur eine kontinuierlich anhaltende Lehrtätigkeit ermöglicht dabei, wirklich suffiziente Erfahrungswerte zu erzielen, die es in der Regel dann erst erlaubt, Lehrtätigkeit signifikant zu optimieren. Während die/der chirurgische HabilitandIn erst dabei ist, sich „Sporen in der chirurgischen Lehre zu verdienen“ (wobei auf mehr oder weniger ausgedehnte Erfahrungen aus Vorjahren der PJ-ler‑, Assistenten- und/oder Stationsarztzeit zurückgegriffen werden kann), sollte die/der Habilitierte durchaus schon auf probate Lehrerfahrungen zurückblicken und diese nutzbringend einbeziehen können.

Einer/m chirurgischen HabilitandIn ist suffiziente und vor allem auch wiederholte Gelegenheit einzuräumen, adäquate chirurgische Lehre so auch in ihrer „Abstufung“ zu leisten, ob (Haupt‑)Vorlesung, Seminar, Blockpraktikum, in der Famulatur oder PJ-ler-Betreuung als auch in der OSCE-Prüfung.

### Anforderungen an die/den HabilitandIn

Die/der HabilitandIn sollte entsprechend der angestrebten Lehrbefähigung zu den Aktivposten in der akademischen, so auch in der medizinischen Lehre zählen. Damit sollten folgerichtig auch die/der chirurgische HabilitandIn in ideeller Vorbereitung, Planung, Organisation, Durchführung, Modifikation und nachhaltiger Etablierung integriert und in vorderer Reihe tätig sein.

Damit ist selbstredend eine Vorbildrolle verbunden.

Dennoch richten sich die spezifischen Anforderungen an die/den HabilitandIn nach der Habilitationsordnung der jeweiligen Universität, wenn auch die der Lehre klar zugewandte Grundhaltung unabdingbar ist.

In aller Regel muss die/der HabilitandIn ein Studium an einer Universität oder einer dieser gleichstehenden Hochschule des In- oder Auslandes mit deutscher Anerkennung des ausländischen Abschlusses absolviert haben. Sie/er muss darüber hinaus zur Führung des dem Studiengang entsprechenden, von einer Hochschule im Geltungsbereich des Grundgesetzes verliehenen Doktorgrades oder gleichwertigen akademischen Grades einer ausländischen wissenschaftlichen Hochschule berechtigt sein. Vor Einreichen des Habilitationsantrages muss die Bewerberin/der Bewerber eine zumeist mehrjährige Lehrtätigkeit an einer wissenschaftlichen Hochschule ausgeübt haben mit nachweisbaren bzw. dokumentierten als auch nachvollziehbaren Eckpunkten geleisteter Lehre. Für ärztliche BewerberInnen, die die Habilitation in einem Gebiet anstreben, für das es in Deutschland eine geregelte Weiterbildung zum Facharzt gibt, sollen die Voraussetzungen dieser Facharztbezeichnung erfüllt sein [4, 5].

In Abhängigkeit der jeweiligen Habilitationsordnung muss die/der HabilitandIn eine festgelegte Anzahl relevanter, möglichst hochrangiger Publikationen, teils in Englisch und getrennt nach Erst- bzw. Senior- oder KoautorInnenschaft, vorweisen. Zudem ist eine Auflistung von gehaltenen wissenschaftlichen und der Fortbildung dienenden Vorträgen und ausgestellten medizinisch-wissenschaftlichen Postern vorzulegen [4].

Deutschlandweit einheitliche Vorgaben erscheinen dahingehend perspektivisch wünschenswert.

Neben dem Verfassen einer Monographie kann die Habilitationsarbeit auch aus einer kumulativen Schrift von Einzelbeiträgen, die einen inhaltlichen Zusammenhang aufweisen und zusammenfassenden bzw. umspannenden Text i.S. eines „roten Fadens“ enthalten sollten, bestehen ([4, 5]; Tab. 1).

**Table 1:** 

Lehrtätigkeit	
Mit Studierenden	In Abwesenheit von Studierenden
Mitgestaltung und Abhaltung von Vorlesungen, Seminaren und Blockpraktika	Konzeption von Lehrplänen, Lehrfällen und Klausurfragen
Betreuung von FamulantInnen und PJ-Studierenden	Aus- und Bewertung schriftlicher Prüfungen
Promotionsbetreuungen	Gremienarbeit mit Teilnahme an der Lehrkommission und der Lehrzirkel
Einbindung von Studierenden in den Operationsdienst	Erstellung chirurgischer Leitdokumente (z. B. einrichtungsspezifische[s] „Chirurgisches Lehrmanual“, Logbücher für das Blockpraktikum und das PJ)
Abnahme mündlicher (Staatsexamens‑)Prüfungen (OSCE, Nachprüfungen etc.)	Ausarbeitung innovativer chirurgischer Lehrinhalte im interdisziplinären Kontext (z. B: „surgery and cancer genetics“; „surgery and microbiology“)
Ggf. Abnahme mündlicher Staatsexamensprüfungen (Ausnahmefall)	Entwicklung innovativer fakultativer Lehrkonzepte (E-Learning, Home-Studying, Lehrfall[präsentation], SkillsLab-assoziierte Themenbreite und Kursprofil, monatliche spezialthemenbasierte Seminare, „bed side teaching“, Lehrveranstaltungen in einer anderen Sprache [insbesondere Englisch], Teilnahme an Dienstbereitschaft, systematische Eingliederung in Operationsteams für Elektiv- [ggf. Notfall-]Eingriffe, Besuch anderer Einrichtungen, fachspezifische Kongressteilnahme – Studierendenprogramm, fachspezifische Journal Clubs etc.)
Prüfungsaufsicht	Innerklinische Weiter‑/Fortbildung von in der Lehre tätigen KollegInnen
Evaluation der Lehrveranstaltungen aus Sicht der Studierenden als auch der Lehrenden	Eigener Besuch lehrunterweisender Fortbildungen
Vermittlung und Erwerb praktischer Fähigkeiten im „SkillsLab“	Abschluss einer lehrdidaktischen Qualifikation
Lehrforschung	Kollegialer interaktiver Kontakt/Austausch mit lehreausübenden KollegInnen und (ggf.) KlinikchefIn
	Aktive zeitnahe Dokumentation eigener Lehraktivität
	Lehrforschung
	Lehrpublikation
	Weiterentwicklung und Etablierung innovativer Lehrformen von Studierenden für Studierende/ein Laienpublikum („science slam“)
	Erstellung eines strukturierten Fragenkatalogs, um die Rolle der HabilitandInnen in der Lehre an chirurgischen, universitären Kliniken zu erheben

*PJ* Pflichtjahr, *OSCE* objective structured clinical examination

Charakterliche Eignung hinsichtlich einer Lehrtätigkeit und Lehrpersönlichkeit, bisherige positive Lehrevaluationen und befürwortende Empfehlungen von KollegInnen und VorgesetztInnen flankieren in geeigneter Weise Habilitationsbestrebungen entsprechend geeigneter KandidatInnen aus Sicht der medizinischen Lehre, was natürlich ebenso für die chirurgische Lehre Gültigkeit aufweist.

Lehrmethodische Ideen und Vorschläge zur organisatorischen Umgestaltung bzw. Optimierung sind willkommen.

Der enge Kontakt zur Habilitationseinrichtung, der (potenziell) die Habilitation gewährenden und das Verfahren durchführenden medizinischen Fakultät/Hochschule und ihren/seinen lehrorganisatorisch tätigen Beauftragten als auch den zahlreich lehrausübenden chirurgischen KollegInnen sowie zum/zur lehrverantwortlichen KlinikchefIn bzw. LehrstuhlinhaberIn erscheint essenziell, vor allem auch hinsichtlich eines interaktiven Anspruchs. Dieser enge Kontakt sollte und kann durch:

- die innerklinische Weiter- und Fortbildung von in der Lehre tätige/n KollegInnen,
- den eigenen Besuch lehrunterweisender Fortbildungen,
- die Ableistung einer lehrdidaktischen Qualifikation (und)
- eine aktive zeitnahe Dokumentation eigener Lehraktivität

erreicht werden.

Nicht zuletzt erscheint die detaillierte Kenntnis und kreative Umsetzung von Inhalten des „Med. Fakultätentages“ und der „Ärztlichen Approbationsordnung“ ratsam.

Aus dem Nachweis der wissenschaftlichen Befähigung i. R. des angestrebten Habilitationsverfahrens erwächst der Anspruch auch eines ureigenen und zu verfolgenden Interesses an der Betreuung wissenschaftlicher Projekte und Themen, z. B. i. R. einer Dissertation.

Über dem Gesamtspektrum an möglichen nutzbringenden Anforderungen steht die Zielstrebigkeit des Kandidaten für das Habilitationsverfahren und eine persönlichkeitsimmanente Konsequenz in der Umsetzung des individuellen „Projekts“ Habilitation.

### Anforderungen ans Umfeld

Der humanmedizinischen Studierendenschaft kommt eine nicht unwesentliche Bedeutung zu, als aufmerksames und interessiertes Auditorium insbesondere in der klinischen Studienphase zu fungieren, um auch durch ein generiertes Echo vor allem der/dem chirurgischen HabilitandIn eine adäquate Rückkopplung zu geben als auch eine kompetente Meinung von StudierendenvertreterInnen zu generieren und in der Lehr- und/oder Habilitationskommission i. R. eines Habilitationsverfahrens zu vertreten.

Nicht zuletzt sollte von Anbeginn für die/den HabilitandIn als auch weiterführend für die/den Habilitierte(n) mit Lehrauftrag von fachlicher Seite für aktive Lehrbetätigung organisatorisch gesorgt werden, um:

- eine bestehende Lehrbefähigung nachhaltig (weiter) zu entwickeln und zu pflegen,
- chirurgische Lehrpersönlichkeiten auftreten und sich vervollkommnen (als auch)
  - sie hinreichende Erfahrungen sammeln zu lassen (und)
- individuelle lehrmethodische Vielfalt zu gewährleisten

bei aller zu verfolgender Wahrung studienplan- und einrichtungsspezifischer Einheitlichkeit und Authentizität.

Verständnis und Förderung durch Dienstvorgesetzte bzw. KlinikchefIn sind dabei in letzter Instanz bedeutsam.

### Chirurgische Lehrtätigkeit mit Studierenden

Die/der HabilitandIn sollte sich obligat in der kurrikularen Lehre engagieren und aktiv Vorlesungen, Seminare und Blockpraktika mitgestalten und natürlich auch selbst aktiv (ab)halten [6]. Zudem ist die Betreuung von FamulantInnen und Studierenden im Praktischen Jahr vorgesehen.

Fakultativ ist die Gestaltung eigener monatlicher Seminar- und Vortragsreihen auch in Anlehnung an das eigene wissenschaftliche Betätigungsfeld (z. B. Inhalt der Habilitationsschrift) sowie eigene klinische und thematische Interessen. Zudem zählen die

- aktive Integration von Medizinstudierenden in den
  - Operationsdienst, insbesondere im Hinblick auf die Einbindung in den Operationsalltag (2. Assistenz bei chirurgischen Eingriffen) (und)
  - Bereitschaftsdienst (sowie)
- individuelle Prüfungsvorbereitung

zur weiteren fakultativen Lehrtätigkeit, derer sich insbesondere HabilitandInnen annehmen sollten.

Es ist hinreichend überliefert, dass gerade eine 1:1(2)-Betreuung dahingehend extrem guten Lehr- und Lernerfolg sichert, aber überdurchschnittliches Lehrengagement erfordert.

Die/der HabilitandIn kann zudem als

- LehrkoordinatorIn (für die chirurgischen Fächer),
- Lehrbeauftragte/r (der vertretenen Fachdisziplin),
- Lehraufsicht (sowie)
- PJ- (oder)
- Famulaturbeauftragte/r

eingesetzt werden.

Die Übergangsphase zwischen „Praktischem Jahr“ und dem sog. „common trunk“ als Teil der chirurgischen Facharztausbildung kann und sollte von der/dem HabilitandIn aktiv mitgestaltet werden [7].

Des Weiteren können Nach- und OSC(P)E(„objective structured clinical [practical] examination“)-Prüfungen mit abgenommen werden [8]. Zudem können praktischen Fähigkeiten im „SkillsLab“ vermittelt werden [9, 10].

Im Ausnahmefall ist auch die Abnahme mündlicher Staatsexamensprüfungen zu befürworten, die allerdings regelhaft eher erfahrenen PrüferInnnen bzw. Habilitierten vorbehalten bleiben sollte.

Die/der HabilitandIn eignet sich zudem wie jeder andere Lehrtätigkeitsausführende für die Evaluation der Lehrveranstaltungen aus Sicht der Studierenden als auch durchaus aus der Perspektive der Lehrenden (Tab. 1).

### Chirurgische Lehrtätigkeit interner und externer HabilitandInnen ohne Studierende

Neben der aktiven Lehrtätigkeit mit Studierenden besteht eine Vielzahl an Möglichkeiten des Engagements in der Lehre ohne Studierende, die sich insbesondere auf organisatorische, planerische, auswertende, evaluierende und methodisch-entwickelnde Aspekte bezieht.

Im Vordergrund stehen hier die Konzeption von Lehrplänen, (interaktiv ausgerichteten) repräsentativen Lehrfällen (über interessante ungewöhnliche Krankheitsbilder, anspruchsvolle Diagnosefindungen, Fallmanagementansprüche und -verläufe), Klausurfragen sowie die Aus- und Bewertung schriftlicher Prüfungen und Lehrpublikationen.

Weitere Gelegenheiten der Mitgestaltung chirurgischer Lehre bestehen in der Teilnahme an Sitzungen der Lehrkommission sowie in der aktiven Mitarbeit in dieser und der Lehrzirkel im Sinne einer Gremienarbeit. Die Tab. 1 gibt hierzu eine Übersicht über die unterschiedlichen Möglichkeiten der Lehraktivität.

Externe und interne HabilitandInnen können – im Sinne der Erstellung chirurgischer Leitdokumente – jeweilige Logbücher für das Blockpraktikum und das Praktische Jahr ausarbeiten (helfen) sowie Lehrinhalte und Tätigkeitsbeschreibungen für die Lehre verschriftlichen und zusammenfassen.

Eine weitere Möglichkeit des Einsatzes in der chirurgischen Lehre besteht in der Ausarbeitung innovativer chirurgischer Lehrinhalte und Lehrkonzepte im interdisziplinären Kontext (z. B: „surgery and cancer genetics“ [11]; „surgery and microbiology“ [12] u. a.). Auch die inhaltliche und strukturelle Konzeption von Lehrformen auf dem Gebiet des E‑Learnings und des Home-Studyings kann zu den Gestaltungsmöglichkeiten externer und interner HabilitandInnen gehören [13, 14].

Vor dem Hintergrund, dass hochrangige Publikationen im Regelfall englischsprachig sind und eine Vielzahl von Studierenden die Möglichkeit sucht und wahrnimmt, im Ausland zu studieren, sollte durch die/den HabilitandIn auch englischsprachige Literatur angeboten oder gar englischsprachig aktiv gelehrt werden.

### Die/der HabilitandIn als wissenschaftliche/r BetreuerIn

Die/der HabilitandIn sollte die Betreuung von Promotionen anstreben. Damit wird zu gegenseitigem Nutzen das Verfolgen individueller oder institutioneller bzw. sogar projektbezogener Forschungsinteressen mit dem Streben nach akademischer Graduierung studierendenseits sinnvoll verbunden. Die/den Habilitierende(n) bereichert es im Sammeln von Lehrerfahrungen auf hohem akademischem Niveau, nicht zuletzt auch zur eigenen wissenschaftlichen und publizistischen Profilierung.

Hierbei ist es denkbar, Studierende bereits in Lehrveranstaltungen für ein Dissertationsthema zu gewinnen. Eine engmaschige Betreuung, bestehend aus regelmäßigen Treffen bzw. zeitnahen Unterredungen *ad libitum*, sollte seitens verlässlicher und vorbildhaft agierender (promotionsbetreuender) HabilitandInnen gewährleistet werden [15]. Neben der „klassischen“ Promotionsbetreuung kann die/der Studierende ermutigt werden, das Promotionsthema auf einem nationalen oder internationalen Kongress in Form eines Vortrags oder eines Posters vorzustellen. Auf diese Weise kann sowohl für den „wissenschaftlichen Nachwuchs“ gesorgt als auch kompetentes Echo zu einem frühen und unmittelbar nutzbringenden Zeitpunkt der Bearbeitung des Dissertationsthemas/-projektes generiert werden [16].

Neben der Promotionsbetreuung sollte die/der HabilitandIn auch offen für die Betreuung intra- als auch extrafakultärer Master- und Bachelorarbeiten sein.

Des Weiteren ist die Integration interessierter und leistungswilliger (Medizin‑)Studierender in wissenschaftliche Journal Clubs, (Jung‑)Forschergruppen oder Teilprojekte unabhängig vom Promotionsanspruch möglich.

### Anforderungen an die/den HabilitandIn im Hinblick auf den Kontakt zu und Umgang mit Studierenden

Um ein erfolgreiches Lehren im Sinne der Vermittlung und letztendlich reliablen Erreichung kurrikularer Lernziele zu gewährleisten, kann ein enger Austausch mit studentischen Organisationen (z. B. institutionell eigener „Fachschaftsrat“) oder in Gremien mit Studierendenvertretung (Lehrkommission) hilfreich sein. Hierbei sollte durch ein hohes Maß an Kommunikativität und Umgänglichkeit der HabilitandInnen gegenseitiger Respekt und ein erfolgreicher thematischer Austausch erzeugt werden.

Studentische Hilfs-(Lehr‑)Kräfte sollten durch die/den HabilitandIn gewonnen und angeleitet werden, um einen suffizienten Unterricht von Studierenden älterer Semester für Studierende jüngerer Semester zu gewährleisten [6].

### Die Rolle der HabilitandInnen in der chirurgischen Lehre in „Abgrenzung“ zu nichthabilitierten ärztlichen KollegInnen

Die Rolle der HabilitandIn in der chirurgischen Lehre kann zu jener von nichthabilitierten ärztlichen KollegInnen (bzw. KollegInnen, die sich nicht zu habilitieren beabsichtigen) abgegrenzt werden.

Aufgrund der geplanten (Sub‑)Spezialisierung durch das jeweilige Habilitationsvorhaben und wegen der gesammelten (administrativen und wissenschaftlichen als auch Lehr‑)Erfahrung im Rahmen dieser wissenschaftlichen Graduierung erscheint die/der HabilitandIn zumeist als geeigneterer, motivierter, kompetenter und interessierter, ja berufener bzw. prädestinierter Promotionsverantwortlicher und -betreuer im Gegensatz zu Kollegen, die sich nicht habilitiert haben oder habilitieren möchten.

Habilitierungsansinnen und Verfolgung der Habilitierungsvoraussetzungen lässt ein vordergründiges Lehrinteresse erwarten – sie stellen eine sich durchaus gegenseitig bedingende Voraussetzung dar.

### Die Rolle der HabilitandInnen in Abhängigkeit medizinischer Disziplinen

Im Vordergrund steht in der vorliegenden Arbeit die/der chirurgisch tätige HabilitandIn. Die Rolle, die in der Lehre eingenommen werden kann, ist durchaus stark fachabhängig. So bestehen die Möglichkeit und die Pflicht, in chirurgischen Fächern neben theoretischem Wissen stets auch kompetente praktische Fertigkeiten zu vermitteln als auch strikt auf ihren anwendungsbereiten Erwerb als Vorbereitung auf die klinisch-chirurgische Praxis zu achten. Auf diese Weise kann die/der chirurgische HabilitandIn wegen des teils mehrstündigen Miteinanderagierens im Operationssaal als intensivere/r BetreuerIn und akademische/r LehrerIn von Studierenden wahrgenommen werden im Vergleich zu möglicherweise ärztlichen KollegInnen nichtoperativer Disziplinen.

Eine nicht unwesentliche Rolle nimmt das obligat staatsexamensrelevante Fach Chirurgie (wie auch die Innere Medizin) ein, womit neben den Chancen einer „ernst genommenen“ fachlichen Verfolgung von Studieninhalten durch die Studierenden eine Beachtung und Aufmerksamkeit gerade der chirurgischen Lehre verknüpft sein dürfte.

Die Breite der chirurgischen Disziplin mit etablierten, komplett autarken Teilfächern sollte aufgrund des immensen Themenprofils und der damit assoziierten nicht unbeträchtlichen Anzahl vorgesehener Lehrstunden die Chancen einer persönlichen (Mehrfach‑)Berücksichtigung für die Abhaltung chirurgischer Lehrveranstaltungen erhöhen.

### Die Rolle der HabilitandInnen in universitären und nichtuniversitäten Krankenhäusern

Die Rolle der HabilitandInnen in der chirurgischen Lehre steht in Abhängigkeit zum Wirkort dieser. In nichtuniversitäten (Lehr‑)Krankenhäusern können die/der chirurgische HabilitandIn die Aufgaben der Lehre insgesamt sehr gut „auf ihre Fahnen schreiben“, so z. B. die komplette bzw. überwiegende PJ- und Famulaturbetreuung organisieren. Hierbei bieten sich regelmäßige wöchentliche Seminare zu praxisrelevanten Themen z. B. auch nach Studierendenwunsch oder von alltägliche Fällen aus der aktuellen klinischen Praxis an. Auch die Promotionsbetreuung für ärztliche KollegInnen (die z. B. als Studierende zuvor in dem entsprechenden Lehrkrankenhaus tätig waren) stellt eine mögliche akademische (Lehr‑)Aktivität an nichtuniversitäten Krankenhäusern dar.

Die Rolle der HabilitandInnen an einer medizinischen Fakultät/einem Universitätsklinikum kann hingegen im höheren Maße geprägt sein von der regelmäßigen Teilnahme an Lehrveranstaltungen aller Art (Praktikum, Seminar, Vorlesung, Problem-orientiertes Lernen [„POL“], „SkillsLab“). Ihre Wahrnehmung sollte für die/den außeruniversitär tätige(n) HabilitandIn auch möglich und realisierbar sein, erfordert jedoch noch mehr Verständnis und Förderung des Dienstvorgesetzten (siehe auch oben „Anforderungen an das Umfeld“).

## Perspektiven und innovative Ansätze

In Zeiten der Globalisierung und Digitalisierung kann die/der HabilitandIn verstärkt den internationalen und interkulturellen Austausch von Studierenden fördern. So sind die ärztlichen KollegInnen vor, während und nach dem Habilitationsvorhaben in aller Regel national und international gut vernetzt. Diese Netzwerke können genutzt werden, um neben einem regelhaften mehrwöchigen studentischen Austausch innovative Lehrformen in Deutschland zu implementieren (Tab. 2).

**Table 2:** 

Perspektiven	Innovative Ansätze
Mitgestaltende Neudefinition des Verhältnisses von Vorlesung, Seminar, Blockpraktikum	Initiierung des internationalen und interkulturellen Austauschs von Studierenden unter Nutzung eigener Netzwerke
Mitvertretende Bewahrung der klassischen Vorlesung (als effektivste Lehrveranstaltung; inkl. Patientenvorstellung etc.) und ihre weiterführende schöpferische Ausgestaltung (Interaktion, TED-Befragung, selektive Aufzeichnung, „Zoom“, ausgewählte themenbasierte Handouts, studierendenbasierte Themenbestreitung, Lehrfilme, Operationsvideos, Liveschaltung in den Operationssaal etc.)	Implementierung innovativer Lehrformen in Deutschland unter Nutzung eigener Netzwerke
Kenntnis und kreative Umsetzung von Inhalten des „Med. Fakultätentages“ und der „Ärztlichen Approbationsordnung“	Lehreinrichtungsspezifische Rekrutierung und Übernahme von Anregungen und Innovationen aus Modellstudiengängen
Individuelle/persönliche Selbstverpflichtung	Entwicklung innovativer fakultativer Lehrkonzepte (Electronic-Learning [E-Learning], Home-Studying, Lehrfall[präsentation], „SkillsLab“-assoziierte Themenbreite und Kursprofil, monatliche spezialthemenbasierte Seminare, „bed side teaching“, Lehrveranstaltungen in einer anderen Sprache [insbesondere Englisch], Teilnahme an Dienstbereitschaft, systematische Eingliederung in Operationsteams für Elektiv- [ggf. Notfall‑]Eingriffe, Besuch anderer Einrichtungen, fachspezifische Kongressteilnahme – Studierendenprogramm, fachspezifische Journal Clubs etc.) – siehe auch Tab. 1
	Mitbewerbung um Lehrpreis als lohnende Zielprämie
	Einbeziehung von Studierenden in die Gestaltung von Kongressen und Tagungen
	(Mitgestaltung und Betreuung der Rolle eines sog. Juniorvorsitzenden im Rahmen von Tagungen – beispielsweise PJ-Studierende, AssistenzärztInnen im ersten Jahr)

*TED* Teledialog, *PJ* Pflichtjahr

**Figure 1:**
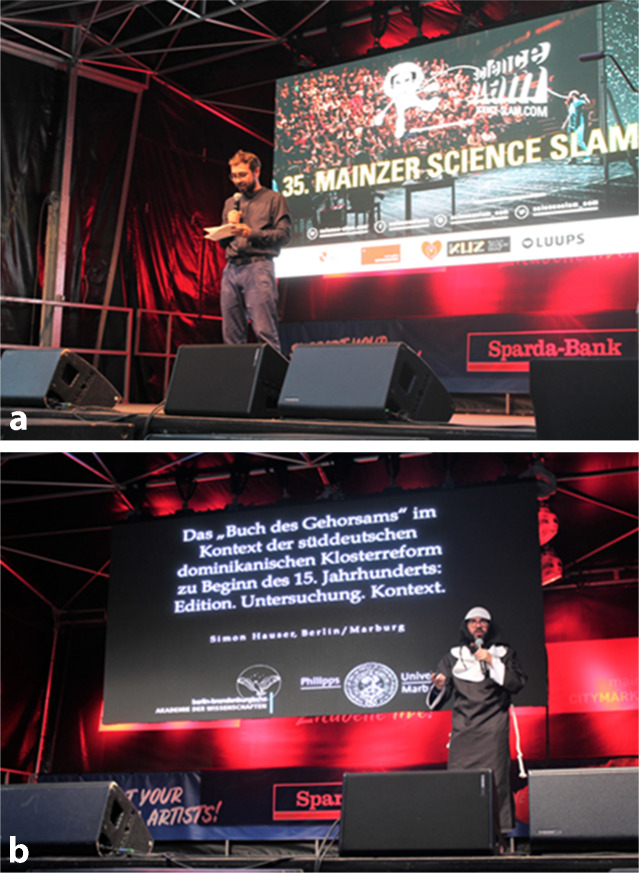

Die/der HabilitandIn könnte Studierende aktiv in die Gestaltung von Kongressen und Tagungen einbeziehen. Auch die Teilnahme an sog. „Science-Slam“-Veranstaltungen, auf denen Promotionsarbeiten vorgestellt werden können, erscheint als ein sehr aussichtsreicher innovativer Lehransatz [17, 18]. Beispielhaft wurde beim „35. Mainzer Science Slam 2020“ eine Vielzahl von Promotionsarbeiten vorgestellt (Abb. 1).

Ferner könnte die Rolle eines/r sog. Juniorvorsitzenden im Rahmen von Tagungen (beispielsweise PJ-Studierende, AssistenzärztInnen im ersten Jahr) von der/dem HabilitandIn aktiv mitgestaltet und betreut werden. Auf diese Weise kann der medizinische Nachwuchs mitgestalten und begeistert werden für diese verschiedenen Fortbildungsformate (Tab. 2).

Seit geraumer Zeit wird durch die Autoren das Konzept verfolgt, studentisch gehaltene Einzelvorlesungen zu implementieren. In fakultativen Seminarveranstaltungen wurde diese Praxis erfolgreich getestet, z. B. in englischer Sprache (Manuskript in Vorbereitung).

Des Weiteren wäre die Durchführung einer Umfrage unter allen universitären chirurgischen Kliniken in Deutschland denkbar, um anhand eines strukturierten Fragenkatalogs die Rolle der/s HabilitandIn in der chirurgischen Lehre im klinischen und Lehralltag zu erfassen (Projektkonzeption in Vorbereitung).

Innovative Ansätze und Ideen erscheinen dabei kaum erschöpflich, sollten aber nach gut bewährter und nutzbringender Einführung in die Lehrpraxis auch Gegenstand und Wert anhaltender Weiterverfolgung sein!

## Diskussion

In der vorliegenden narrativen Kurzübersicht wurde das vielfältige Aufgabenfeld der HabilitandInnen in der chirurgischen Lehre umrissen. Dabei stellt sich die Frage, ob die Habilitation (noch immer) eine lohnende akademische Graduierung darstellt. Ein Pro-Argument kann die Vielfalt des Betätigungsfeldes sein. Habilitierte sind dazu angehalten zu lehren, zu forschen und klinisch sowie administrativ tätig zu werden. Ein abgeschlossenes Habilitationsverfahren kann zudem die Karrierechancen steigern. So ist die abgeschlossene Habilitation die Grundvoraussetzung zur Erlangung einer außerplanmäßigen (apl‑)Professur. In *Wissenschaft als Beruf* kann Max Weber (1912) mit folgendem Satz zitiert werden:

Nur durch strenge Spezialisierung kann der wissenschaftliche Arbeiter tatsächlich das Vollgefühl, einmal und vielleicht nie wieder im Leben, sich zu eigen machen: hier habe ich etwas geleistet, was dauern wird [3].

Der bekannte deutsche Soziologe und Ökonom Max Weber (1864–1920) zeigt hier eine aus eigener Sicht treffende Motivation auf, die Habilitation anzustreben.

Ein Kontra-Argument besteht natürlich in der Tatsache, dass das o. g. Betätigungsfeld auch ohne abgeschlossenes Habilitationsverfahren suffizient sowie mit Freude und Engagement durchgeführt werden kann. Der Blick in die Vereinigten Staaten von Amerika zeigt, dass auch ein Habilitationsverfahren – an deren Stelle tritt vergleichsweise a.e. der Ph. D. („philosophical doctorate“; [19, 20]) – ein leistungsfähiges Lehr- und Wissenschaftssystem etablieren lässt. Letztlich scheint sich die Frage, ob die Habilitation eine lohnende akademische Graduierung ist, nicht wirklich objektiv beantworten zu lassen.

Kritisch anzumerken ist die Tatsache, dass die Habilitation (und auch die apl-Professur) durchaus als „Mittel zum Zweck“ zur Erlangung weiterführender Positionen in der klinischen Hierarchie (Oberarzt/-ärztinnenposition, Chefarzt/-ärztin etc.) dienen kann. Passend dazu ist leider gelegentlich zu beobachten, dass sich das wissenschaftliche Engagement nach abgeschlossener Habilitation und/oder erlangter apl-Professur bei einigen KollegInnen leider in Grenzen hält. Hier wäre eine konkrete Verpflichtung, zu forschen und zu publizieren, denkbar. Wiederum daran könnte die Zuweisung von abzuhaltenden Lehrveranstaltungen gekoppelt werden, die für das Beibehalten der jeweiligen Graduierung notwendig ist.

Die wirtschaftlichen Ziele universitärer und nichtuniversitärer Kliniken und die damit zumeist verbundenen Streichungen von Planstellen können aus eigener Erfahrung zu einer höheren klinischen Arbeitsbelastung führen. Es ist denkbar, dass dieser Umstand die Qualität der Lehre alteriert. So kann in der Zusammenschau die Frage aufgeworfen werden, ob die Fusion der Tätigkeitsbereiche Lehre, Forschung und Patientenversorgung, die durch die/den HabilitandIn verkörpert wird, noch zeitgemäß ist. Eine Möglichkeit besteht natürlich in einer schärferen Trennung zwischen Forschung, Lehre und klinischer Arbeit. Diese kann durch höhere Vorgaben an eine Habilitation (Publikationsleistung, Einwerben hoher Drittmittelbeträge, obligate Forschungsaufenthalte etc.) – zuletzt beispielsweise erfolgt an der medizinischen Fakultät der Universität Zürich [21] – erreicht werden. Aus eigener Sicht ist dies jedoch nicht anzustreben, da eine Vielzahl wichtiger wissenschaftlich-klinischer Fragestellungen auf den (langjährigen) Erfahrungen klinisch tätiger ÄrztInnen beruhen, denen die Möglichkeit gegeben werden sollte, sich weiterhin habilitieren zu können. Eine Lösung dieses Konflikts besteht u. a. in (dem Politikum) der suffizienteren Finanzierung medizinischer Fakultäten [21], z. B. in einer angezeigten und fast schon überfälligen Vergütung von Lehraktivitäten als auch Forschungsleistungen in angemessener Weise.

Als eine der Stärken dieser Übersicht wird das originelle Thema angesehen, das bisher nur eine ungenügende Beachtung fand und in der Gesamterschließung chirurgischer Lehre eigentlich nicht fehlen darf. Obwohl sich durchaus thematische Aspekte mit anderen Fächern überschneiden, gibt es fachspezifisch-chirurgische, die diese Abhandlung vom Anspruch her insbesondere herauszustellen suchte.

Als Limitierung der vorliegenden Arbeit ist das eher niedrige Evidenzniveau zu nennen. Nach eigener Kenntnis existieren keine Studien, die die Rolle der HabilitandInnen in der chirurgischen Lehre näher untersuch(t)en und darstellen. Die Mitverfassung des Textes durch Lehrende unterschiedlicher chirurgischer Kliniken hätte zudem etwaige zusätzliche Aspekte, die hier (möglicherweise) nicht bedacht wurden, mit einbezogen. So wäre nicht zuletzt der Einbezug von Lehrenden an Universitäten mit Modellstudiengängen hilfreich.

## Schlussfolgerung

Die/der HabilitandIn spielt in der chirurgischen Lehre eine zentrale Rolle und sollte Aktivposten und Vorbild sein. Ein breites Aufgabenspektrum mit Abhaltung und Gestaltung von Vorlesungen, Seminaren, Blockpraktika bis hin zu direkter studentischer Betreuung im Rahmen der Famulatur, des Praktischen Jahres und der Promotionsbetreuung sowie fakultative Lehrveranstaltungen auch sehr individuellen Zuschnitts und innovativer Prägung kann von der/vom HabilitandIn suffizient erfüllt werden, ist wünschenswert, ja berufenermaßen nicht zuletzt aufgrund der Habilitationsabsichten zu fordern.
